# Benchtop comparison of seven ureteroscopes: evaluating physical properties and deflection with flexible and navigable suction access sheaths

**DOI:** 10.1111/bju.70124

**Published:** 2025-12-28

**Authors:** Koushikk S Ayyappan, Richard Menzies‐Wilson, Amir Mashia Jaafari, Hasan Al‐Sattar, Ben Turney

**Affiliations:** ^1^ Nuffield Department of Surgery University of Oxford Oxford UK; ^2^ School of Medicine and Biomedical Sciences University of Oxford Oxford UK

**Keywords:** deflection, endourology, FANS, flexible and navigable suction access sheaths, flexible ureteroscopes, irrigation, ureteroscopes

## Abstract

**Objectives:**

To evaluate the physical properties of seven ureteroscopes (URSs) and compare the maximal angle of deflection (MAD) when used with flexible and navigable suction access sheaths (FANSs) of varying sizes.

**Materials and Methods:**

Seven commercial URSs (ranging from 6.3 to 9.5 F in size) were evaluated for outer diameter, irrigation flow rate, image resolution, colour reproduction and MAD. MAD was measured under three conditions: standalone URS deflection without the FANS; standard deflection of the FANS while positioned at the URS tip; and advanced FANS deflection, with the URS fully deflected beyond the FANS, and the FANS advanced. For each URS type, standalone deflection was repeated five times, and FANS deflections were repeated four times to calculate an average. FANS sizes of 10/12 F, 11/13 F and 12/14 F (ClearPetra) were tested.

**Results:**

The HugeMed URS had the smallest scope diameter (6.3 F) and the lowest flow (20 mL/min), while the Endoso URS had the highest flow (32 mL/min). All the URSs had similar resolutions except the MacroLux, Seegen and Endoso URSs, which were noticeably superior in this respect. Colour reproduction was best with the MacroLux and Endoso URSs. Without a FANS, the standalone mean MAD across all URS types was 293°. Standard deflection with FANS significantly decreased the MAD (up to a 49% reduction), whereas advanced deflection maintained the MAD (up to 269°). Larger FANS, especially the 12/14‐F size, tended to reduce deflection. The MacroLux URS maintained the highest MAD across all FANS sizes, followed by the Seegen and Urotech devices.

**Conclusion:**

Ureteroscope deflection significantly varied by model. Use of a FANS reduced deflection angles, especially with larger sheaths. However, advancing the FANS over a deflected scope preserved deflection angles. Overall, the MacroLux URS showed the best deflection with FANS, whereas the Seegen, Endoso and Urotech URSs showed a balance between flow rate, optics and deflection. These findings could inform clinicians in their selection of a URS for endourology procedures.

**Abbreviations**
FANS: flexible and navigable suction access sheaths
MAD: maximal angle of deflection
SFR: stone‐free rate
URS: ureteroscope

## Introduction

Flexible ureteroscopy has evolved over the last few decades and is now among the mainstay treatments for many upper urinary tract conditions, including kidney stones and urothelial carcinoma [1, 2]. Many different commercial ureteroscopes (URSs) are available for clinical use [3], with varying designs and characteristics.

Flexible and navigable suction ureteric access sheaths (FANSs) are a recent development in endourology [4] and feature a flexible tip and suction port to help remove stone fragments. They have a lumen through which the URS is passed, and their use is correlated with high stone‐free rates (SFRs) [5, 6]. URS deflection, crucial for accessing difficult anatomical regions such as the lower pole calyces [7], has been reported to be limited whilst using a FANS [8]; therefore, the choice of scope and FANS is important in maximising utility and efficacy. However, there is limited evidence on how they interact [4, 5, 8].

We aimed to compare the physical characteristics of seven different commercially available URS types, including size, irrigant fluid flow rate, image quality (colour reproducibility and resolution), and how the maximum angle of deflection (MAD) of the URS changes when used with FANS of varying sizes.

## Materials and Methods

Benchtop testing was performed on the following URSs: HugeMed 6.3 F (HU30M); Urotech 7.5 F (URS‐7.5S‐R); MacroLux 7.5 F (CoralView®); Pusen 7.5 F (PU3033AH); Seegen 7.5 F (UV‐US130‐H); Endoso 7.5 F (PUR12‐4); and Boston Scientific 9.5 F (BSC; LithoVue). Only one URS from each manufacturer was tested. Three sizes of ClearPetra FANS (10/12 F, 11/13 F and 12/14 F) were used.

### Diameters and Lengths

The outer diameters of the URS were measured with callipers and the inner diameter was measured by inserting ‘engineers fractional drill bits in 0.1‐mm increments’ into the lumen. Working length was obtained from the manufacturer's specification for each URS.

### Optics Characterisation

Each URS was connected to its respective monitor, and an image was captured under ambient light with optimised scope brightness.

Image resolution was evaluated using the 1951 U.S. Air Force Test Pattern Card (Edmund Optics, USA) at a 2‐cm distance (similar to previous papers [9]). Colour reproducibility was evaluated at 10 cm using a subjective image of a rainbow colour spectrum. For each scope, two images were captured – one for resolution and one for colour.

### Flow Rates

Irrigation flow rates were measured by attaching a 500‐mL saline pressure bag to the URS, at a standard height of 1 m to generate flow. After allowing the system to equilibrate for 1 min, inflow irrigation pressure was set to 100 mmHg using a Watson Marlow IP31 peristaltic pump, with pressure monitored on an OMEGA inline pressure monitor (PXM409‐USBH). Flow rate was measured with the tip of the URS undeflected, and a 200‐μm Olympus laser fibre was inserted at 1 mm beyond the tip to simulate lasertripsy conditions. The volume of water emptying from the URS over 30 s was measured with a syringe. Three trials were performed, and the average flow was calculated as the mean volume per min.

### Deflection Angles

The MAD values for each URS were measured under three conditions: (i) standalone deflection, with the URS deflected without a FANS; (ii) standard deflection, where the FANS tip is aligned at the URS proximal end, and the URS fully deflected inside the FANS; and (iii) advanced deflection, with the URS fully deflected beyond the FANS tip, before the FANS is advanced over the URS until its proximal end is aligned with the URS.

Standard and advanced deflection are shown in Fig. S1. Images were captured from directly above, and the angles were determined using a protractor. A 200‐μm Olympus laser fibre was inserted into each URS to simulate lasertripsy conditions. To reduce bias resulting from the FANS becoming more flexible with repeated use, we rotated the URS by 180° in the FANS between repeats. Deflection was performed on both sides of the scope.

For standalone deflection, five trials were performed, and for the other conditions, four trials. The mean deflection angle was calculated.

### Statistics

All data were analysed using prism graphpad version 10. For flow rates and MAD, one‐way ANOVA, followed by Tukey's post hoc test was performed for increased statistical power (as *n* ≤ 5 for each experiment). For comparison of mean MAD values across all URSs, the unpaired *t*‐test was used. *P* values < 0.05 were taken to indicate statistical significance and all tests were two‐sided.

## Results

### Physical Dimensions

The physical dimensions of the URS are summarised in Table 1. Notably, the HugeMed URS had the smallest scope diameter, at 6.3 F, while the BSC had the largest, at 9.5 F. All other URSs had a diameter of 7.5 F. The URS tip outflow designs are shown in Fig. S2, and the handles and connectors are shown in Fig. S3.

**Table 1:** Summary of physical dimensions of seven ureteroscopes.

Ureteroscope	Outer diameter, F	Working length, cm	Working channel diameter, mm
HugeMed (HU30M)	6.3	65	1.2
Urotech (URS‐7.5S‐R)	7.5	70	1.2
MacroLux (CoralView)	7.5	68	1.2
Pusen (PU3033AH)	7.5	65	1.2
Seegen (UV‐US130‐H)	7.5	70	1.2
Endoso (PUR12‐4)	7.5	67	1.2
Boston Scientific LithoVue (BSC LV)	9.5	68	1.2

### Irrigation Flow Rates

We compared the flow rates through each URS at 100‐mmHg irrigation pressure, as this varies with the diameter and designs of the working channel.

**Fig. 1:**
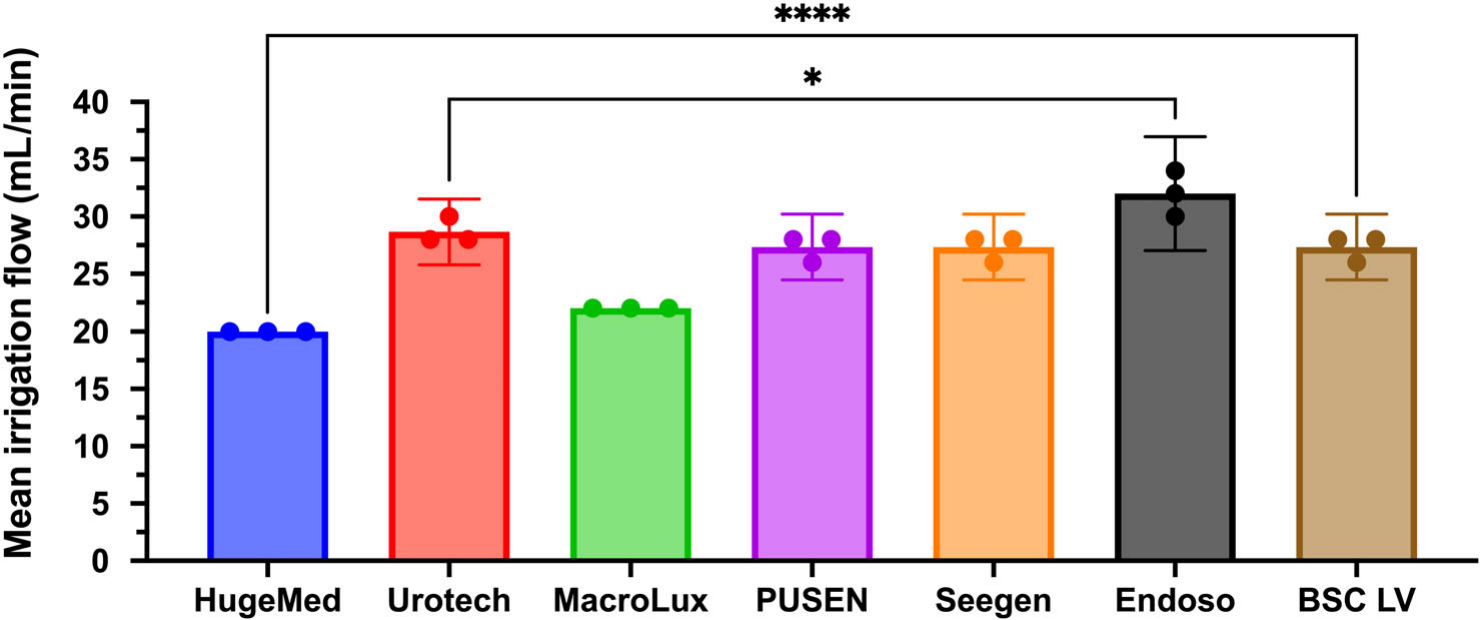
Mean irrigation flow rate in seven ureteroscopes (URSs). Irrigation pressure of 100 mmHg was used, and a 200‐μm laser fibre was placed into each URS lumen. *n* = 3 for each group. Data shown as mean ± 95% CI. *P ≤ 0.05; ****P ≤ 0.0001.

As shown in Fig. 1, at the same irrigation pressure, the HugeMed URS had the lowest flow rate, at 20 mL/min, as expected given the smaller diameter of its working channel. This was comparable to the flow rate for the MacroLux URS (*P* < 0.05). The BSC URS had a flow of 27.3 mL/min, significantly greater than that of the HugeMed device (*P* < 0.0001). However, the Endoso URS had the highest flow rate, at 32 mL/min, exceeding the next best, Urotech, which was measured at 28.9 mL/min (*P* = 0.0403).

### Optical Characteristics

**Fig. 2:**
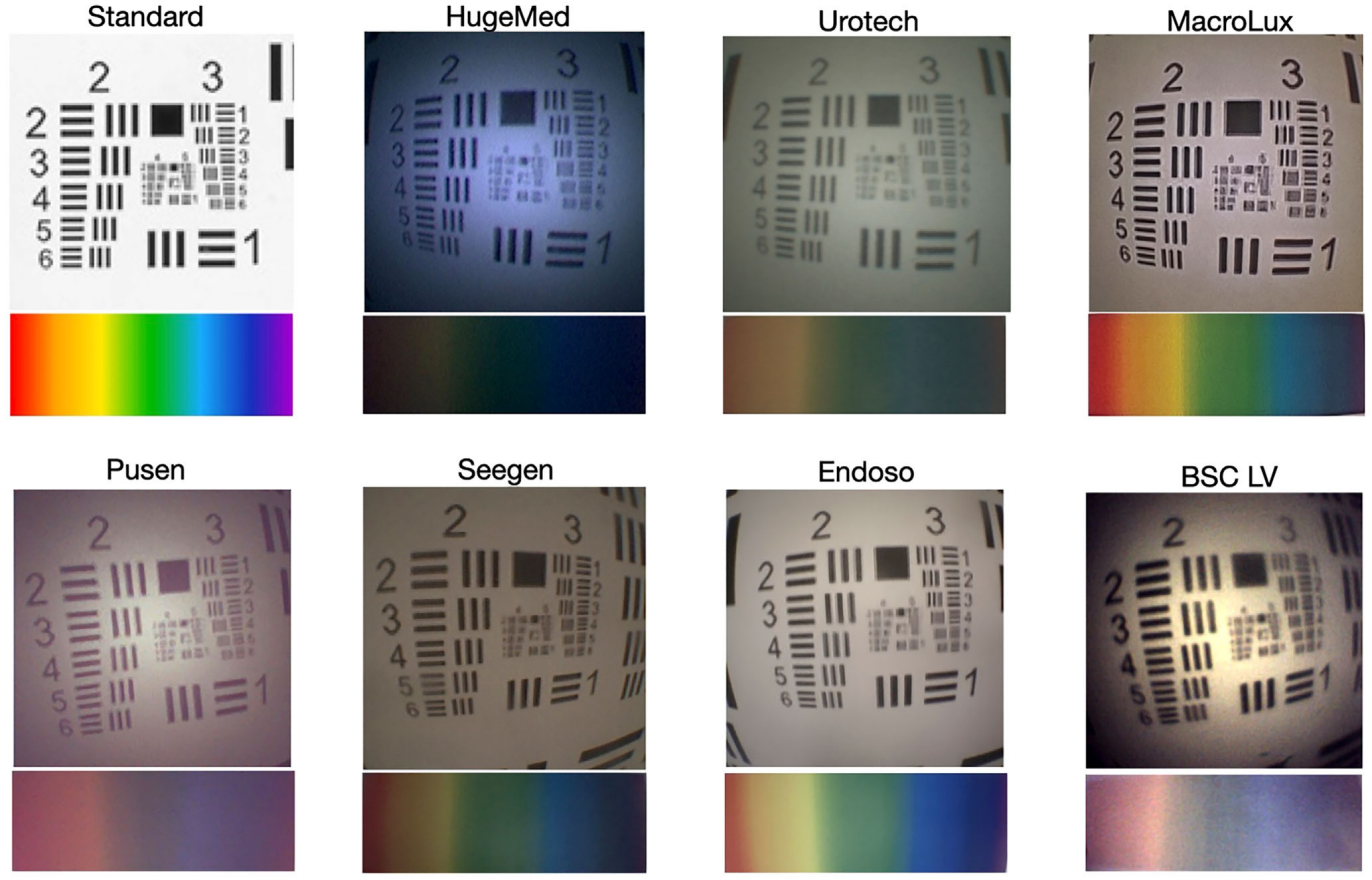
Optical characteristics of seven ureteroscopes. Image resolution was assessed at a 2‐cm distance using the 1951 USAF Test Pattern Card. Colour differentiation used a subjective rainbow spectrum at 10 cm. Standard images are shown for comparison. *n* = 1.

We also subjectively assessed the optical characteristics of the URS (Fig. 2). Resolution was consistently high, with superior images observed for the MacroLux, Endoso and Seegen URSs. Colour reproducibility was generally weak for all URSs; however, Endoso and MacroLux demonstrated the brightest and clearest colours.

### Maximum Angle of Deflection

**Fig. 3:**
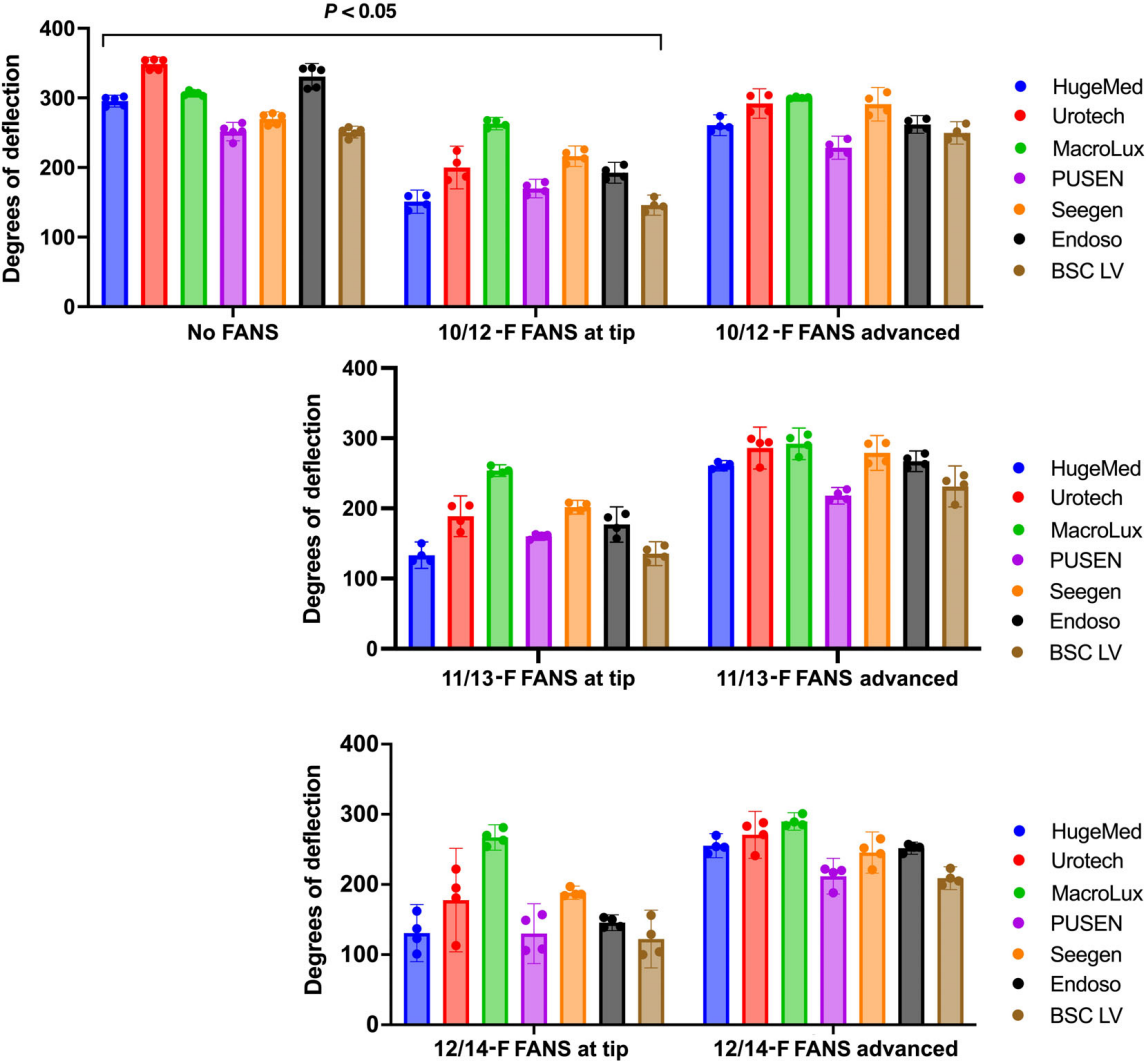
Maximum deflection angles for each ureteroscope (URS). Conditions were: standalone URS without a flexible and navigable suction access sheath (FANS); with a 10/12‐F FANS located at the tip; with the 10/12‐F FANS advanced over the deflected scope; with a 11/13‐F FANS at the tip; with a 11/13‐F FANS, advanced deflection; with a 12/14‐F FANS at the tip; and a 12/14‐Fr FANS, advanced deflection. No FANS: *n* = 5; other groups: *n* = 4. Data shown as mean ± 95% CI.

In addition, we tested the MAD across all URSs, with different FANS diameters (Fig. 3). The mean standalone MAD across all URS was 293° ± 38°.

### Deflection Technique

Under standard deflection with the 10/12‐F FANS, compared to standalone MAD, the mean MAD fell by 35%, to 190° ± 41° (*P* < 0.05 for all URSs). Standard deflection of the 11/13‐F and 12/14‐F sheaths also showed a decrease relative to the standalone condition, with a mean MAD of 179° and 166°, respectively (*P* < 0.05 for all URSs).

Advanced deflection with the 10/12‐F and 11/13‐F FANS mitigated the reduction in MAD. With the 10/12‐F FANS, relative to standalone, the mean MAD fell by 8% to 269° ± 26°. With the 11/13‐F FANS, it fell by 11% to 262° ± 28°. These results were not statistically significant (*P* = 0.21 and *P* = 0.108), indicating minimal performance loss. However, advanced deflection of the 12/14‐F FANS did not mitigate the reduction in MAD compared to the standalone condition, and there was still a 15% fall to 248° ± 29° (*P* = 0.026).

### FANS Size

Larger FANS sizes tended to decrease mean MAD across all URSs. For standard deflection, the mean MAD across all URSs tested fell from 191° ± 41° with the 10/12‐F FANS, to 179° ± 42° with the 11/13‐F FANS, and further to 166° ± 51° with the 12/14‐F FANS. However, this difference was not statistically significant (*P* = 0.583). A similar trend was seen for advanced deflection (*P* = 0.364).

When comparing specific URS types, there was a notable difference – especially between the 10/12‐F and the 12/14‐F devices. Under standard deflection, the 12/14‐F FANS showed a lower MAD relative to the 10/12‐F FANS for the Pusen (23%, *P* = 0.0071), Seegen (13%, *P* = 0.0448) and Endoso URSs (24%; *P* < 0.0001). With advanced deflection, reductions were seen with both the Seegen (16%, *P* = 0.0004) and the BSC URSs (16%, *P* = 0.0079).

### Inter‐URS Comparison

Under standalone deflection, the Urotech and Endoso URSs had the highest MADs, at 349° and 331°, respectively. The HugeMed (295°) and MacroLux URSs (306°) showed similar MADs (*P* = 0.49). The Pusen, Seegen and BSC URSs had the lowest MADs, ranging from 250° to 270°. This shows no clear link between URS size and MAD.

Additionally, there was no link between URS size and MAD when using a FANS. Under standard deflection with the 10/12‐F FANS, the MacroLux URS had the highest MAD at 263° (*P* < 0.05), followed by the Seegen, Urotech and Endoso URSs, which had similar MADs (193°–216°; *P* > 0.05). Finally, the Pusen, HugeMed and BSC URSs had the lowest MADs, and these were comparable (146°–170°; *P* > 0.05). This pattern was consistent across 10/12‐F FANS in the advanced deflection condition, and for the 11/13‐F and 12/14‐Fr groups, regardless of deflection technique.

## Discussion

There are many URS brands on the market, each with different features. Understanding and comparing these differences could help surgeons to select the most suitable URS. In this benchtop study, we compared the physical properties of seven types of URS, and their angles of deflection across three different FANS sizes.

Image quality is crucial during laser lithotripsy. One factor which affects this is inflow rate into the URS. Higher flow rates improve visibility [10], but excessive flow can increase intrarenal pressure and cause backflow issues [10]. In our study, the highest flow rates were seen with the 7.5‐F Endoso, Urotech, Seegen and PUSEN URSs and the 9.5‐F BSC URS, while the 6.3‐F HugeMed and the 7.5‐F MacroLux URSs had the lowest flow rates (with the same irrigation pressure). Although there is a trend towards smaller URS sizes [3] – due to their potentially lower associated risk of ureteric damage [11], reduced intrarenal pressures [12], and higher success rates without ureteric dilatation [13] – our findings suggest that smaller diameters may limit irrigation flow rate.

Flow rate is not solely determined by channel diameter, however, since scopes with the same diameter showed different flow rates. Scope design, including channel geometry [14], lumen, and tip shape, may also affect inflow resistance [15] and therefore flow rate. Furthermore, in clinical practice, modern fluid management systems can partially compensate for higher URS inflow resistance by increasing driving pressure and automatically maintaining a target flow rate [16, 17], enhancing visibility. However, compensatory increases in driving pressure can result in intrarenal pressure exceeding 15–30 mmHg, which is associated with backflow and sepsis [18] (especially since some fluid management systems have been shown to underestimate pressure whilst overestimating flow rate and temperature [19]). Therefore, our benchtop measurements of flow rates and inflow resistance remain clinically relevant for guiding safe and effective irrigation.

Additional factors affecting image quality are resolution and colour reproduction. We observed considerable variability in both, with colour reproducibility generally being suboptimal. These findings indicate that improving colour reproduction may be an important focus for future URS development.

Flexible and navigable access sheaths have been shown to significantly improve SFRs and postoperative complications, relative to standard access sheaths [20]. Choosing the right URS–FANS combination is important for navigating difficult anatomy, for example, the pelvi‐ureteric junction [7], and for aspiration, maintaining irrigation, avoiding injury, and improving SFRs [4]. In our study, sheath advancement over the scope resulted in a MAD of 248°–269°, significantly greater than standard deflection. This is consistent with the only other known study on this topic, which reported a MAD range of 218°–277° [8].

We also noted a trend towards reduced MAD when FANS sheath size is increased for certain URS types, such as the Seegen URS. This is consistent with several studies in the literature [8, 21, 22]. It is known that the 10/12‐F FANS is between 20% and 40% more flexible than the 12/14‐F FANS [8, 22]. A conference paper reported a similar negative correlation between FANS diameter and deflection [23], and deflection loss was even more pronounced with the 13/15‐F FANS, which had an average deflection of 215° [23]. This would support the clinical use of smaller FANS sizes, especially since larger sheaths may increase the risk of ureteric injury [11].

Nevertheless, some evidence supports the use of larger FANS sizes. A multicentre study involving 295 patients found that larger sheaths were subjectively reported to offer improved visibility and suctioning of larger stone fragments [24], especially in the lower pole, consistent with previous findings [22]. However, as anatomy such as infundibulopelvic angles were not considered in this study, no further inference can be made.

When comparing MADs across URS types, no clear relationship was observed between scope size and deflection. For instance, under both standalone and standard FANS deflection, the 9.5‐F BSC and the 6.3‐F HugeMed URS showed similar MAD values. For standalone deflection, the 7.5‐F Urotech and Endoso URSs had the highest MAD, whereas for standard FANS deflection, the 7.5‐F MacroLux and Seegen performed better. Studies in the literature are also conflicting [8, 9, 25, 26]. One study comparing the 7.5‐F and 9.5‐F Pusen URSs found that the 7.5‐F device had lower deflection [26], while another reported that a 7.5‐Fr OTU URS had the highest deflection among scopes ranging from 7.2 F to 9.1 F in size [25]. These findings suggest that URS deflection angle may be influenced more by design than size.

This study had several limitations. Firstly, as a benchtop experiment, it may not fully replicate *in vivo* renal anatomy [7]. This includes individual variation, collecting system capacity and compliance, and the impact of blood and dust – all of which can affect flow rates, optics and deflection. Secondly, only one URS from each manufacturer was tested; therefore, inter‐scope variability within each manufacturer could not be assessed. Additionally, we only repeated each experiment four to five times, which was adequate for our primary objectives but may have lacked the sensitivity for detecting more subtle differences (e.g. a reduction in mean MAD across all URS with larger FANS sizes). Thirdly, our optical characterisation of colour and resolution were subjective, with an *n* of 1 for each scope; however, there is no ideal method of quantification.

Future work could focus on comparing irrigation flow rate and visibility with different URS–FANS combinations and whether this changes with an automated irrigation system [19]. Additionally, measuring the resistance and force required to deflect the URS lever may provide further insights, as we hypothesise this changes with URS type and FANS size. Finally, pressure‐sensing sheaths are a novel innovation for real‐time intrarenal pressure monitoring, and contain a pressure‐measuring port [27]. Future experiments could investigate whether this affects bending stiffness [28] and reduces MAD. A comparable effect may also occur with a pressure‐sensing URS [29].

In conclusion, URSs differ in design and deflection capability, making the choice of URS and FANS important for effective navigation within the pelvicalyceal system. We characterised seven different types of URS, and found that irrigation flow rates were lower with the HugeMed and MacroLux URSs, and resolution and colour reproduction were best with the MacroLux, Endoso and Seegen URSs. Use of FANS reduced deflection angles, especially with the larger sizes of 11/13 and 12/14 F. The MacroLux URS showed the greatest and most consistent deflection with FANS, followed by the Seegen and Urotech URSs. We demonstrated that advancing the FANS over a deflected scope improved deflection and may be the optimal technique.

## Funding

The author(s) received no financial support for the research, authorship, and/or publication of this article.

## Disclosure of Interests

Nothing is declared.

## Supporting information


**Figure S1.** Demonstration of flexible and navigable suction access sheaths (FANS) deflection techniques. (A) Standard FANS deflection with the ureteroscope (URS) at tip of the sheath. (B) Advanced deflection – the URS is already fully deflected before the FANS is advanced over the scope.

**Figure S2.** Images of seven ureteroscope tips.

**Figure S3.** Images of seven ureteroscope handles and connector types.
