# Supplementary figures and images for: Benchtop comparison of seven ureteroscopes: evaluating physical properties and deflection with flexible and navigable suction access sheaths
Source: BJU Int. 2025 Dec 28;137(Suppl 3):S86–92. doi: 10.1111/bju.70124 (PMC12950932; doi:10.1111/bju.70124)

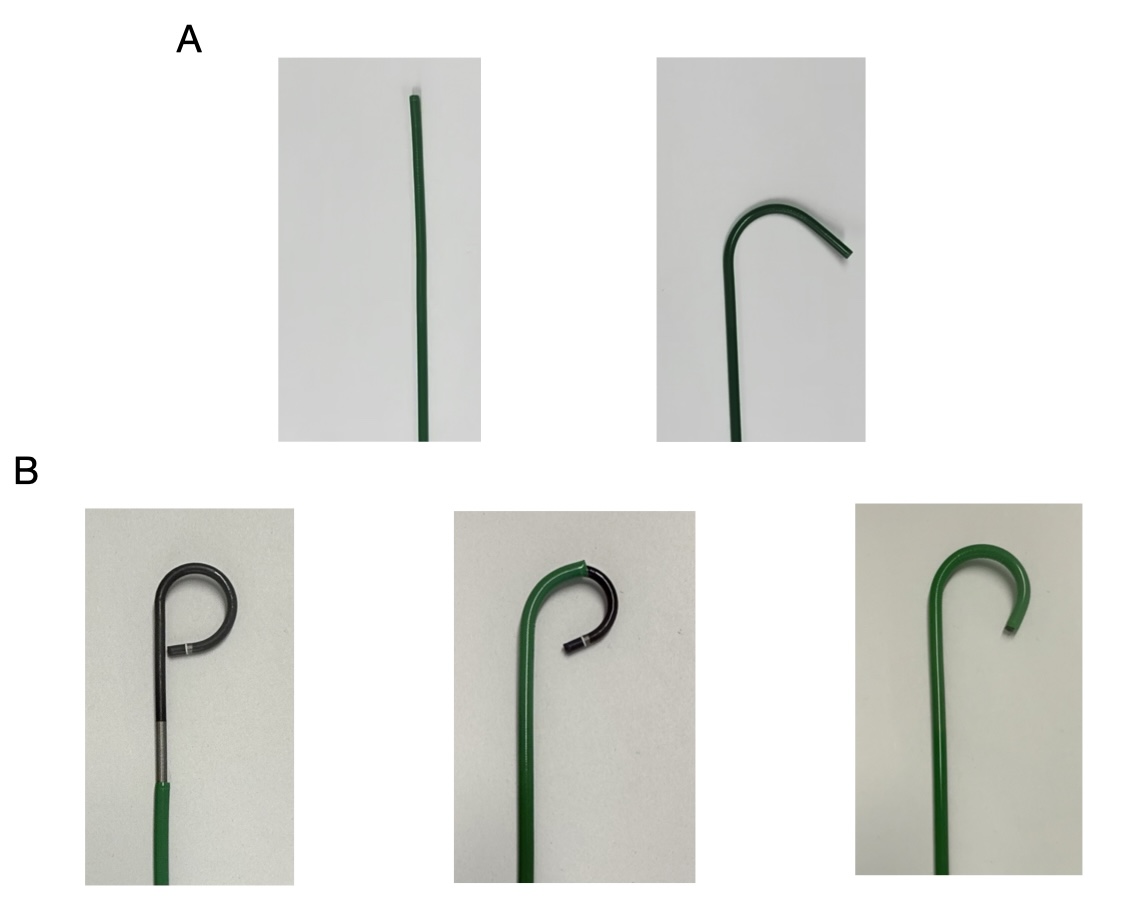

Supplement: Supplementary file 1 — Figure S1. Demonstration of flexible and navigable suction access sheaths (FANS) deflection techniques. (A) Standard FANS deflection with the ureteroscope (URS) at tip of the sheath. (B) Advanced deflection – the URS is already fully deflected before the FANS is advanced over the scope. [file BJU-137-S86-s002.jpeg]

HugeMed

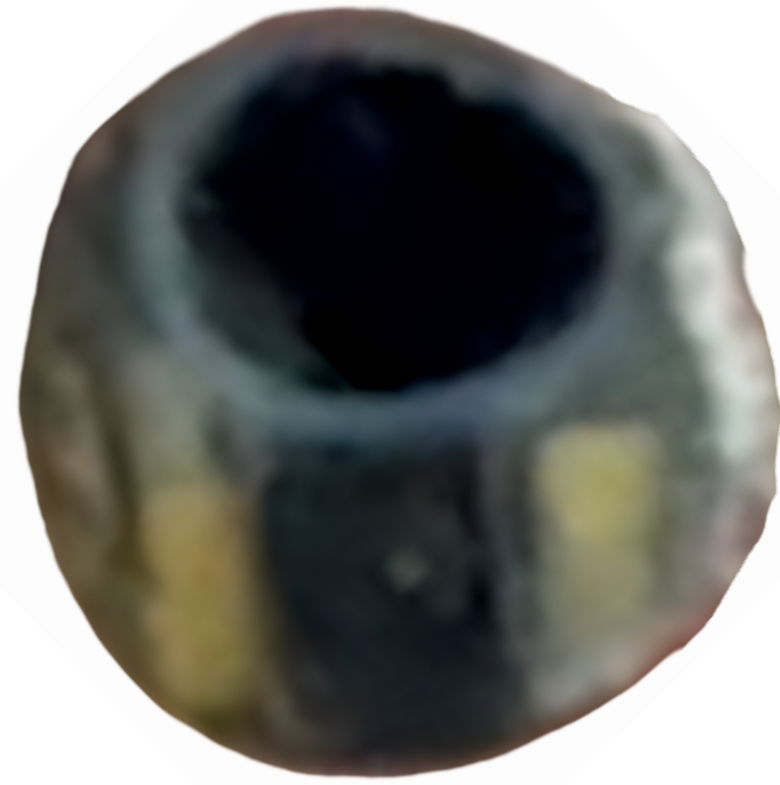

Urotech

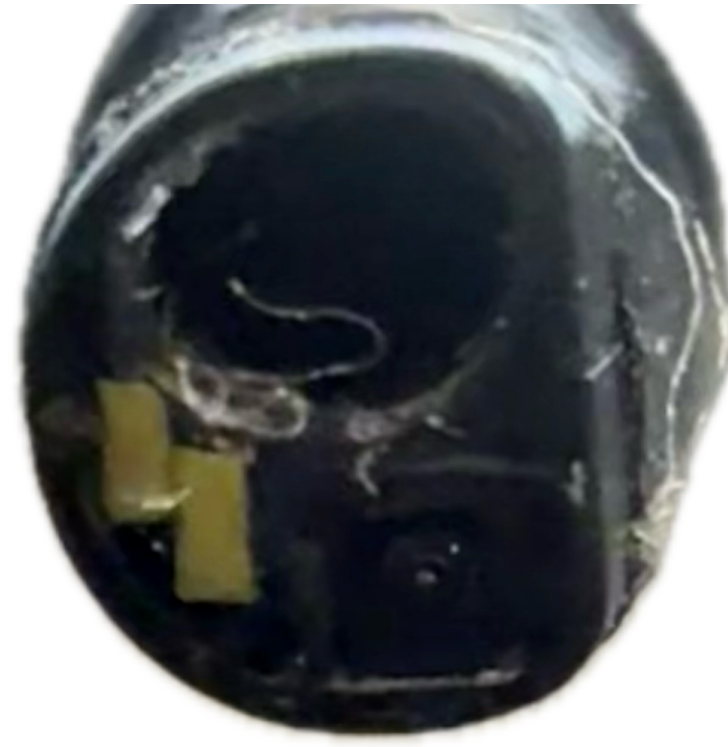

MacroLux

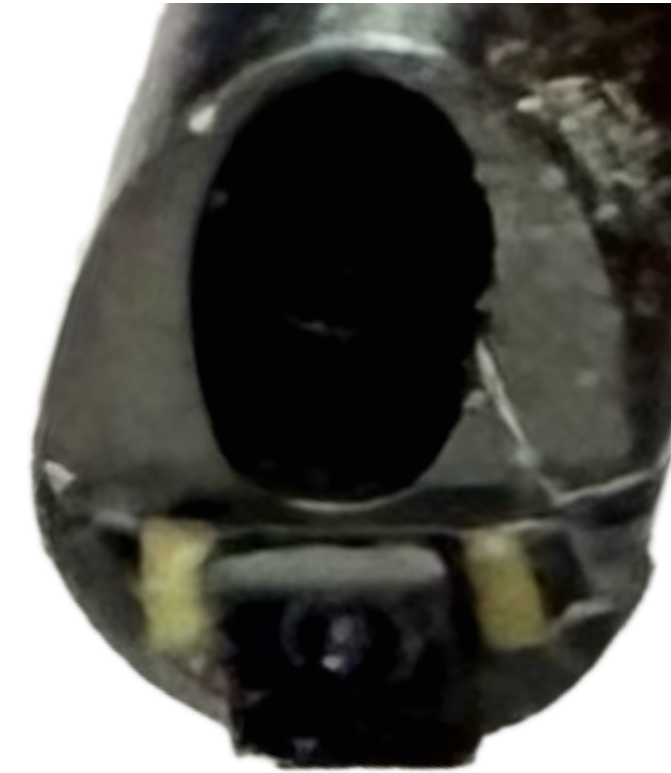

Pusen

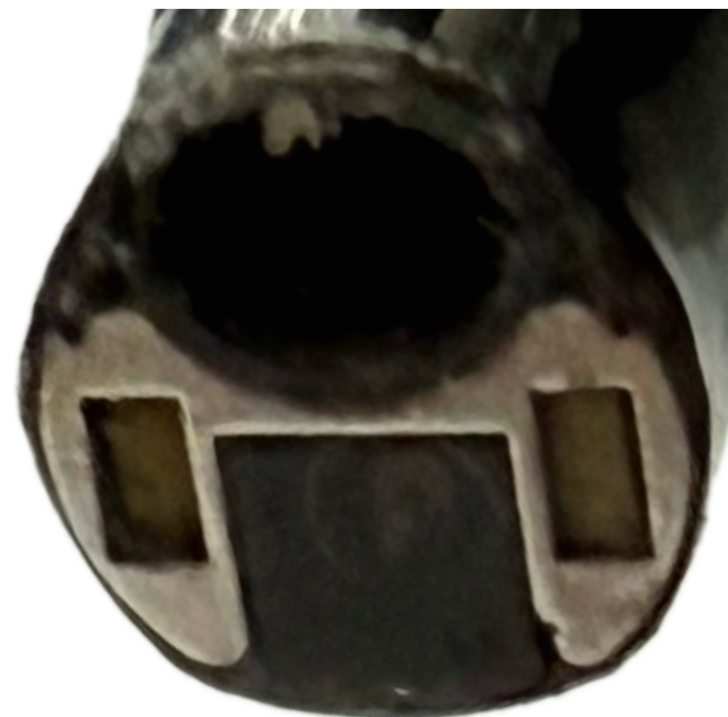

Seegen

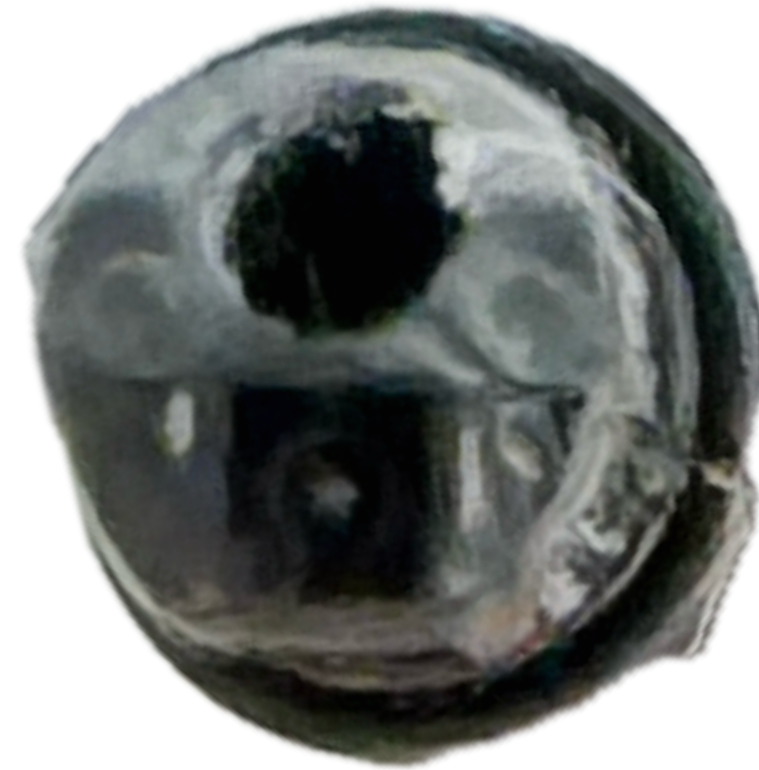

Endoso

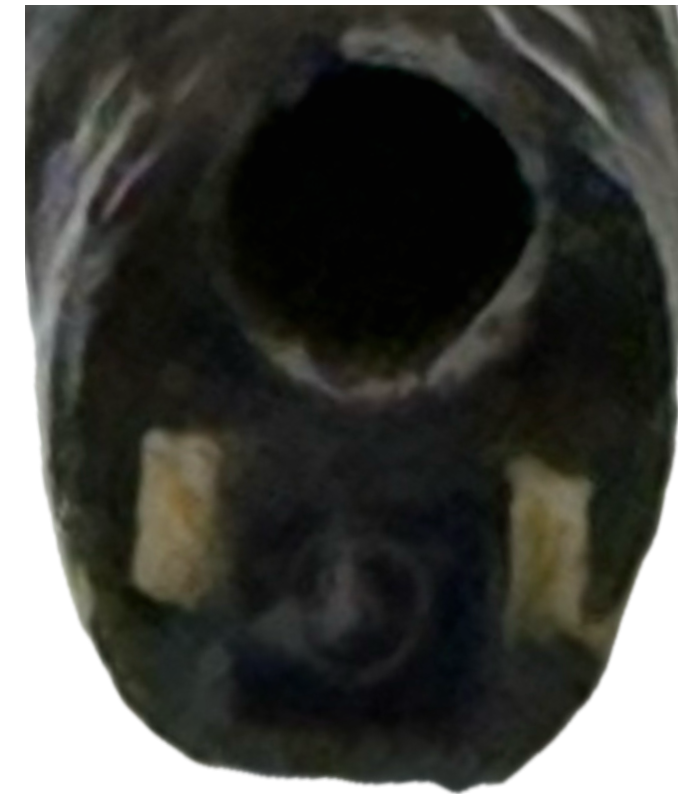

BSC LV

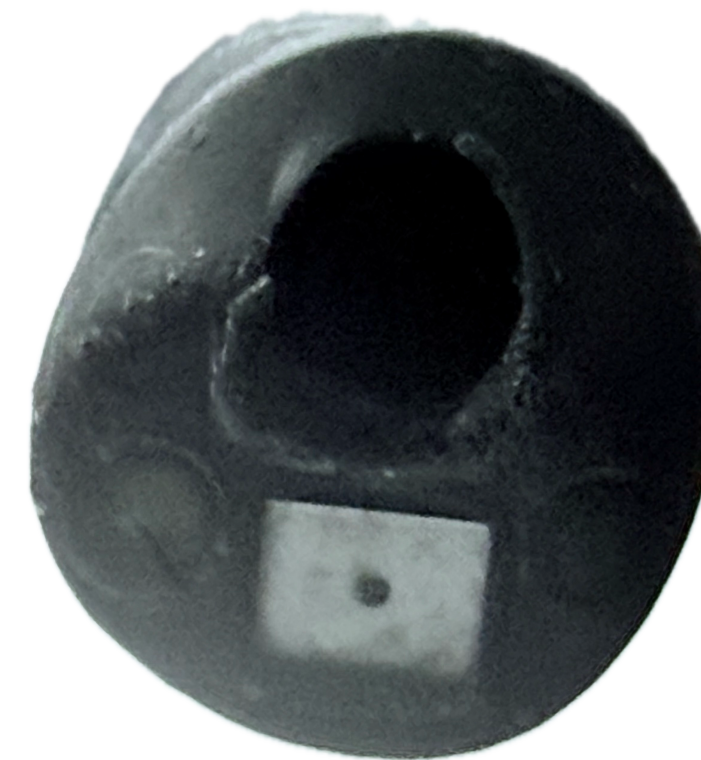

Supplement: Supplementary file 2 — Figure S2. Images of seven ureteroscope tips. [file BJU-137-S86-s003.pdf]

HugeMed

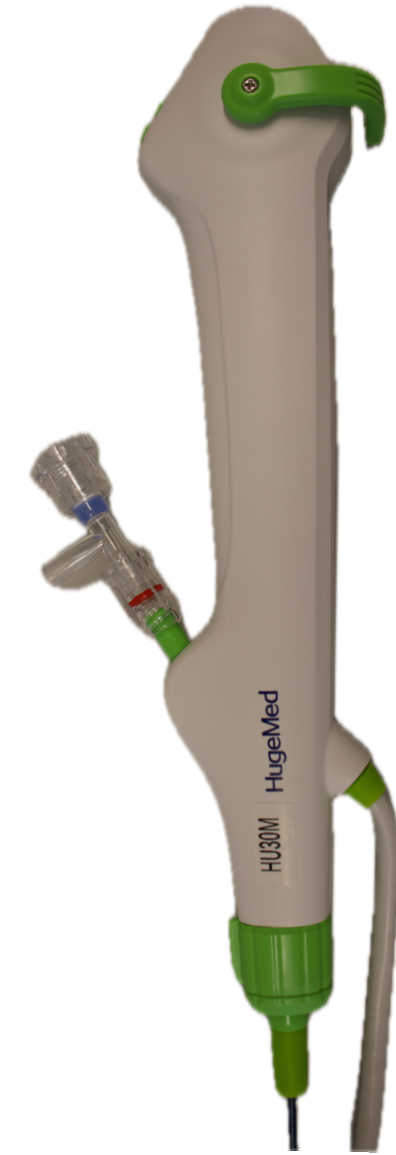

Urotech

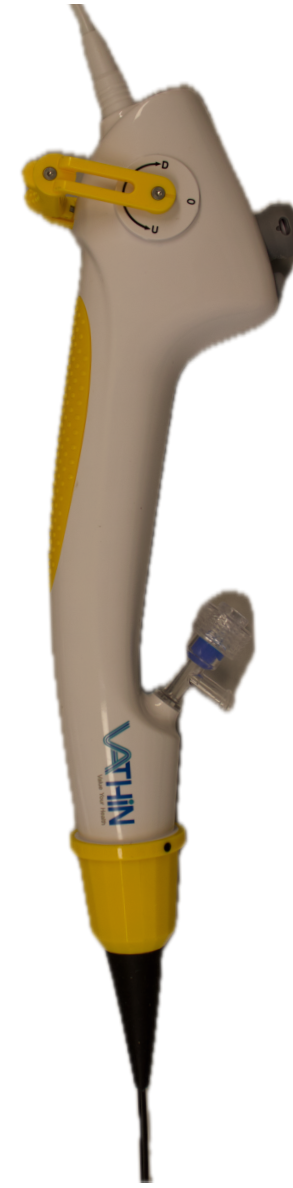

MacroLux

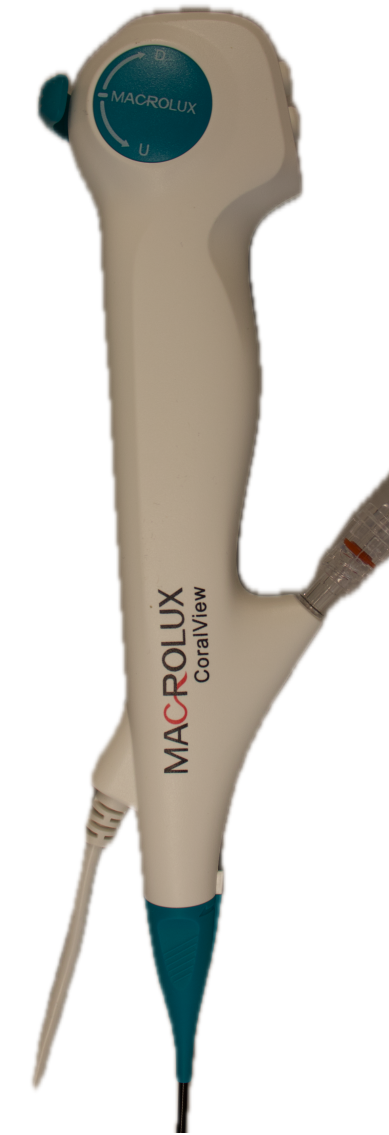

Pusen

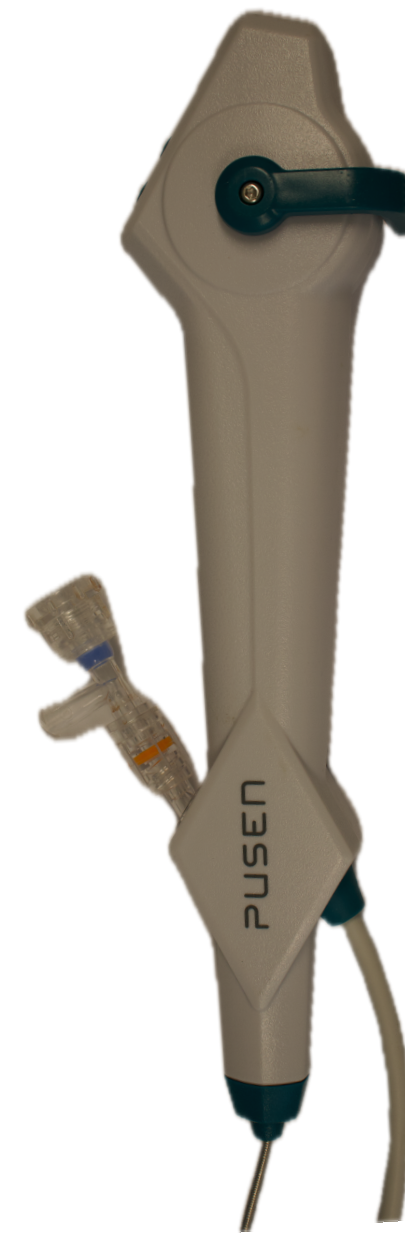

Seegen

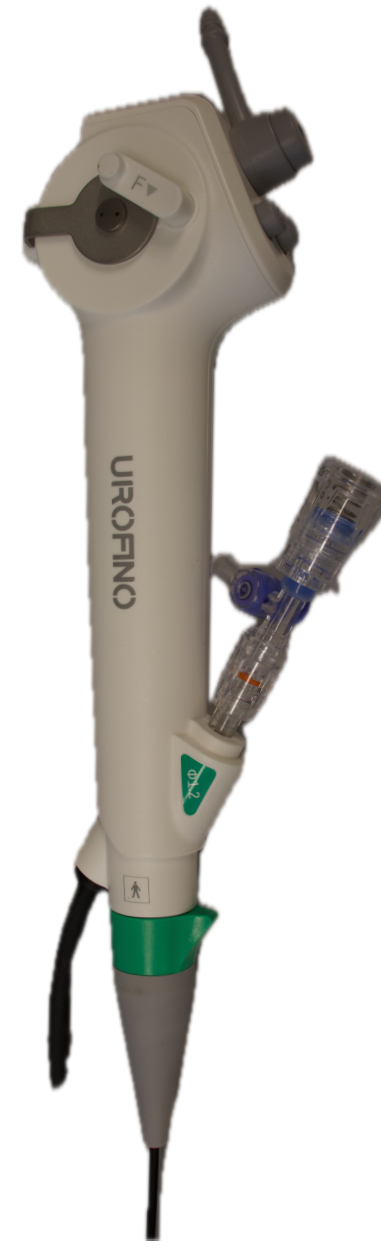

Endoso

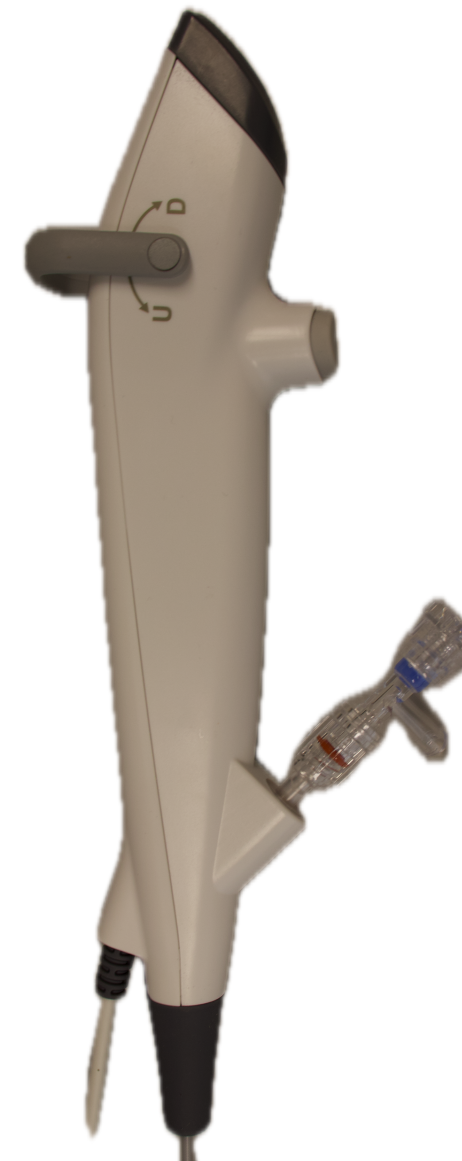

BSC LV

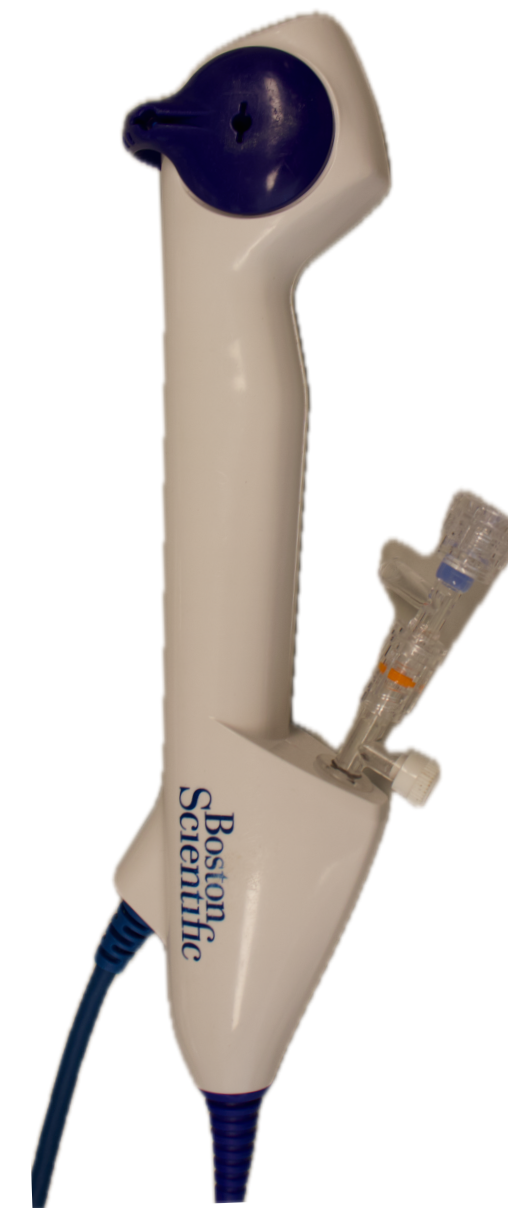

Supplement: Supplementary file 3 — Figure S3. Images of seven ureteroscope handles and connector types. [file BJU-137-S86-s001.pdf]
